# Device-to-Device Aided Cooperative Relaying Scheme Exploiting Spatial Modulation: An Interference Free Strategy †

**DOI:** 10.3390/s20247048

**Published:** 2020-12-09

**Authors:** Md. Shahriar Kamal, Md. Fazlul Kader, S. M. Riazul Islam, Heejung Yu

**Affiliations:** 1 Department of Electrical and Electronic Engineering, University of Chittagong, Chittagong 4331, Bangladesh; srksmaran786@gmail.com; 2 Department of Computer Science and Engineering, Sejong University, Seoul 05006, Korea; riaz@sejong.ac.kr; 3 Department of Electronics and Information Engineering, Korea University, Sejong 30019, Korea

**Keywords:** bit error rate, device-to-device communication, interference free communications, spatial modulation, spectral efficiency

## Abstract

In this paper, a novel interference free dual-hop device-to-device (D2D) aided cooperative relaying strategy (CRS) based on spatial modulation (SM) (termed D2D-CRS-SM) is proposed. In D2D-CRS-SM, two cellular users (e.g., a near user (NU) and a relay-aided far user (FU)) and a pair of D2D transmitter (D${}_{1}$)-receivers (D${}_{2}$) are served in two time-slots. Two different scenarios are investigated considering information reception criteria at the NU. Irrespective of the scenarios, the base station (BS) exploits SM to map information bits into two sets: modulation bits and antenna index, in phase-1. In the first scenario, the BS maps FU information as the modulation bits and NU information as antenna index, whereas modulation bits correspond to NU information and the antenna index carries FU’s information in scenario-2. The iterative-maximum ratio combining (*i*-MRC) technique is then used by NU and D${}_{1}$ to de-map their desired information bits. During phase-2, D${}_{1}$ also exploits SM to forward FU’s information received from BS and its own information bits to the D2D receiver D${}_{2}$. Then, FU and D${}_{2}$ retrieve their desired information by using *i*-MRC. Due to exploiting SM in both phases, interference free information reception is possible at each receiving node without allocating any fixed transmit power. The performance of D2D-CRS-SM is studied in terms of bit-error rate and spectral efficiency considering *M*-ary phase shift keying and quadrature amplitude modulation. Finally, the efficiency of D2D-CRS-SM is demonstrated via the Monte Carlo simulation.

## 1. Introduction

### 1.1. Technological Background

Cellular communication is an essential part of the daily activities of billions of people. The number of cellular users are increasing exponentially. The existing radio resources are congested heavily because of continuously emerging applications and services. Therefore, more effective techniques are required to efficiently use the scarce radio resources [1,2]. Furthermore, existing wireless technologies such as fourth generation (4G) is unable to fulfill the ever-growing capacity demands by the wireless users. As a result, a new wireless communication technology termed fifth-generation (5G) [3,4] has started to be deployed in the different parts of the world. However, a single technology alone cannot fulfill the performance requirements of 5G and beyond networks; multiple technologies should be integrated [5,6]. This motivates us to exploit the spatial modulation (SM) in a cooperative relaying scenario with the aid of D2D communication to achieve interference free communication, the key work of this paper.

In a situation where the communication link between the source and destination becomes hostile for data transmission due to poor channel condition or fading in the channel (e.g., user located at cell edge), the use of cooperative communication techniques is a viable solution. In cooperative relaying strategy (CRS), the source transmits information data to the destination which is also heard by a relay node being somewhere in between the source and the destination. Because of its location, both source-to-relay and relay-to-destination channel conditions become favorable for reliable communications. Then the relay eventually processes and forwards the overheard data signal to the destination following some cooperation protocols. With the received signal from the relay, the destination decodes the information transmitted from the source [7,8,9]. Cooperative communication potentially improves network coverage and link reliability while reducing cost [10,11].

In recent years, wireless researchers around the globe have investigated various advanced communication paradigms, including SM multiple-input and multiple-output (MIMO) (termed SM-MIMO) to enhance the capacity and reliability performance of the system or network with reduced complexity [12,13,14,15,16,17,18]. In MIMO, multiple antennas transmit and receive at the same time using conventional modulation techniques that results in a capacity gain [19]. However, the performance of MIMO communications largely depend on antenna spacing of transmission and reception [20,21]. It also depends on antenna synchronization among the transmit antennas and the interference cancellation techniques used to reduce the inter-channel interference (ICI) at the receiver [22,23]. SM-MIMO is promising to solve these issues. Unlike MIMO, SM allows only one antenna to transmit at a particular time instant while other antennas remain silent. This in turn avoids ICI and interference cancellation techniques are therefore not required at the receiver. Furthermore, there is no correlation among the transmit antennas and no synchronization among them is required. Thus, SM-MIMO offers reduced receiver complexity [12,13,14,15,16,17,18].

Conventional modulation techniques such as *M*-ary phase shift keying (*M*-PSK) and *M*-ary quadrature amplitude modulation (*M*-QAM) map a certain number of information bits into a symbol. In SM, the two dimensional signal plane is extended to a new dimension called spatial dimension. The spatial domain is an outcome of the idea of using the antenna numbers as an additional source of information. Therefore, information bits are mapped not only into the transmitted symbol but also into an actual physical location of the antenna. The number of data bits that can be transmitted using SM-MIMO depends upon the constellation diagram used and the number of transmit antennas. To better understand this mapping, the following example is given. Suppose that five data bits can be mapped into 16-PSK and two transmit antennas. However, if the channel condition is not compatible with 16-PSK, the same data rate can be achieved by a combination of 8-PSK and four transmit antennas. Due to the expansion of modulation to the spatial domain, higher number of data bits is transferred by the transmitted symbol while the symbol duration remains the same. As a result, improved spectrum efficiency is achieved [12].

Every communication in a cellular network took place through the base station (BS) till 4G cellular networks and no direct communication was allowed between two devices under licensed spectrum. However, direct communication between devices using licensed cellular band is expected to be allowed in 5G systems which is called device-to-device (D2D) communication technology. D2D communication allows devices to communicate with each other without or limited involvement of a BS [24,25,26,27,28,29]. As D2D users can directly communicate among themselves using the same cellular infrastructure, it not only offloads traffic from the core network but also improves applications and services offered by the system. Additionally, higher bit rates are achieved along with reduced energy consumption.

Furthermore, D2D communication enables better connectivity and signal strength for cell edge or congested area users. Nevertheless, there exists a high susceptibility of interference between the D2D and cellular communication, since D2D users share licensed cellular frequency resources with the cellular users. Therefore, efficient power control, selection of communication mode, and efficient resource allocation are important issues for interference management in cellular network with D2D communications [30,31].

### 1.2. Related Works

In recent years, due to its advantageous unique characteristics, adopting SM in cooperative communications has received considerable attention from researchers [32]. To provide improved system performance, cooperative SM is applied in different areas such as cooperative relaying [32,33,34,35,36], spectrum sharing [37,38,39], wireless security [40,41], energy harvesting [42,43] and so on.

In [32], a cooperative scheme is proposed based on SM (termed CoSM) to mitigate the inter-cell interference experienced by the cell edge user. A multi-cell cellular network is considered where cells are sectored by rearranged fractional frequency reuse. Additionally, a three-tier cellular architecture is presented since extra amount of feedback is required to exploit CoSM to enhance the quality of service for cell edge mobile stations. In [33,34], SM-based half-duplex CRS are proposed, where a dedicated relay equipped with single antenna forwards source information to destination exploiting amplify-and-forward (AF) strategy. At the destination, maximum likelihood detector is used to estimate antenna and symbol information. The performance of [33] is studied over independent Rayleigh fading channel, whereas spatially correlated Rayleigh Channel is considered in [34]. In [35], a joint bit-to-symbol mapping scheme is proposed in a cooperative SM system for both AF and decode-and-forward (DF) relaying techniques to elevate the performance of the system by using the cosine similarity among the single-antenna relay nodes and channel state information (CSI) of each transmit antenna. A dedicated relay-aided cooperative SM system is proposed in [36] for two common relaying protocols AF and DF, considering multiple antennas at all nodes.

In [37], a cooperative spectrum sharing protocol based on overlay paradigm is proposed for cognitive radio networks (CRNs), where primary transmitter (PT) is equipped with single-antenna. A secondary transmitter (ST) exploiting SM assists the PT to communicate with the primary receiver (PR) in exchange of an opportunity of spectrum sharing. A cooperative spectrum sharing protocol for a CRN is proposed in [38] considering multiple antennas at all nodes. SM is adopted by both PT and ST, where ST acts as a DF relay to forward primary information and its own information simultaneously. In [39], a cooperative spectrum sharing protocol employing orthogonal SM is presented, where both PT and ST transmit simultaneously on the same frequency channel.

Considering the eavesdropping vulnerability of wireless communication, the information-theoretic secrecy performance of an SM-based cooperative AF relaying system adopting destination-based- jamming is investigated in [40]. In [41], the physical layer spoofing attack is studied for a dual-hop cooperative communication network that exploits SM. Considering the whole CSI is known to the spoofer, the investigation points out that the spoofing attack can extensively damage SM-based cooperative communication. A cooperative full-duplex DF relaying radio frequency energy harvesting protocol using time-switching architecture is proposed in [42]. A dual antenna relay exploiting SM harvests energy from the received source signal, where source is equipped with single antenna. In [43], a wireless-powered communication strategy is presented where SM is used to elevate system achievable rate. A wireless device adopts SM to simultaneously communicates with an information receiver and harvests radio frequency energy from a wireless energy transmitter. SM allows self-energy recycling at wireless device through partial energy harvesting from the energy that is used for information transmission. However, none of these works considered D2D communications in the proposed network scenarios [32,33,34,35,36,37,38,39,40,41,42,43].

D2D communication in cooperative communication scenarios are studied extensively in the literature without exploiting SM [44,45,46,47,48]. A comprehensive overview of the cooperative D2D techniques is presented in [44] along with future research directions. A brief discussion on different types of simulators including Vienna long term evolution-advanced is also provided. The performance of a D2D aided cellular downlink system is studied in [45], where a D2D user cooperatively relays the traffic of a cellular far user (FU) and transmits its signal to the D2D receiver simultaneously exploiting non-orthogonal multiple access. Two different types of D2D aided cooperative schemes for cellular uplink transmission system are proposed in [46], where D2D agents are selected randomly by the BS based on certain density. The performance of a cooperative cellular uplink network is investigated in [47], where a D2D transmitter acts as a cooperative relay for a cellular user and transmits its signal to the D2D receiver simultaneously. To maximize the achievable sum data rate, optimal power allocation coefficient and spectrum allocation indicator are also identified. Social aware cooperative D2D networks are investigated in [48]. Moreover, formation of social relationship, cooperative network evaluation based on social learning, and peer selection on the basis of social relationship are also discussed.

Furthermore, from the literature [25,45], it is apparent that each user is assigned a specific frequency band, time slot, or code in order to avoid inter-symbol or inter-user interference in orthogonal multiple access. In contrast, the main idea of non-orthogonal multiple access (NOMA) is that multiple users share the same physical resources (e.g., frequency, time, and code) by allocating different amounts of power to the different users. Therefore, it is clear that although same physical resources are shared by NOMA users, each user must be allocated a different amount of power to exploit successive interference cancellation at the receiving nodes to decode the corresponding symbols.

Unlike the existing works, this study proposes a dual-hop D2D aided CRS exploiting SM, where two cellular users (e.g., a near user (NU) and a relay aided FU) and a pair of D2D transmitter (D${}_{1}$)-receiver (D${}_{2}$) are served in two cooperative phases (e.g., phase-1 ($P_{1}$) and phase-2 ($P_{2}$)). Each phase requires one time-slot. The BS transmits data bits for NU and FU at the same time exploiting SM in $P_{1}$. FU, located at cell edge, has poor channel condition with low signal-to-noise ratio (SNR). D2D capable device D${}_{1}$ forwards FU information along with its own data for D${}_{2}$ using SM in $P_{2}$. While keeping all the benefits of cooperative D2D communication, the proposed scheme make the system interference free because of the inherent characteristics of SM. Moreover, there will be no leasing of time slots or power allocation for both cellular and D2D communication.

### 1.3. Contributions

The major contributions of this work are summarized as follows:

- A novel D2D aided two-phase CRS exploiting SM-MIMO (termed D2D-CRS-SM) is proposed, where interference cancellation and management techniques are not required at any of the communication nodes. Two different scenarios are investigated considering information reception criteria at NU during $P_{1}$.
- The proposed protocol demonstrates how interference free information reception is possible at multiple cellulars and D2D users in the same frequency band considering downlink transmission scenario. Moreover, unlike other popular method such as NOMA, no fixed portion of transmitted power is required to be allocated for a user.
- In conventional SM [12], both symbol and antenna indices of a transmitter are the desired information of a single receiver. However, in D2D-CRS-SM, symbol and antenna indices are the desired information of two different receivers; the protocol, thus, facilitates multi-user communication.
- The performance of D2D-CRS-SM is analyzed and investigated, in terms of bit error rate (BER), spectral efficiency (SE), and sum SE considering *M*-PSK and *M*-QAM over independent Rayleigh fading channels. Moreover, comparative results with Vertical-Bell Laboratories layered space-time (VBLAST) spatial multiplexing MIMO technique [49] with zero-forcing (ZF) detector [50] (termed VBLAST-ZF) is also provided.
- The efficiency of D2D-CRS-SM is validated via Monte Carlo simulation. It is observed that sum SE in both scenarios is almost identical for similar set of parameters.

### 1.4. Organization

**Figure 1:**
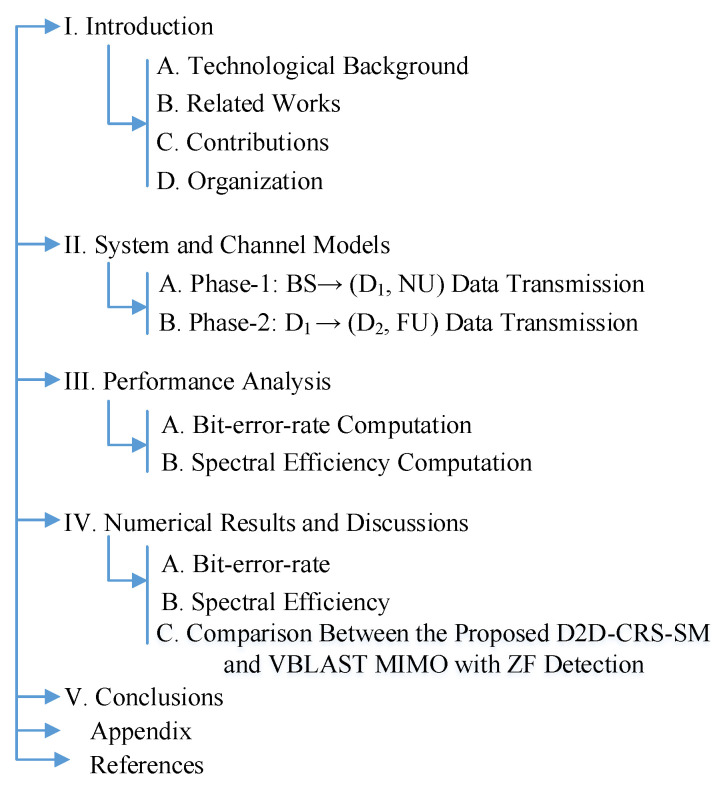
The organization of the article.

The rest of the paper is organized as follows. The system and channel models of the proposed cooperative relaying scheme are described in Section 2. In Section 3 the performance evaluation of the proposed cooperative protocol is formulated for different users. The simulation and analytical results are presented in Section 4. The final section concludes our work. To look at a glance, Figure 1 presents the organization of this article.

## 2. System and Channel Models

**Figure 2:**
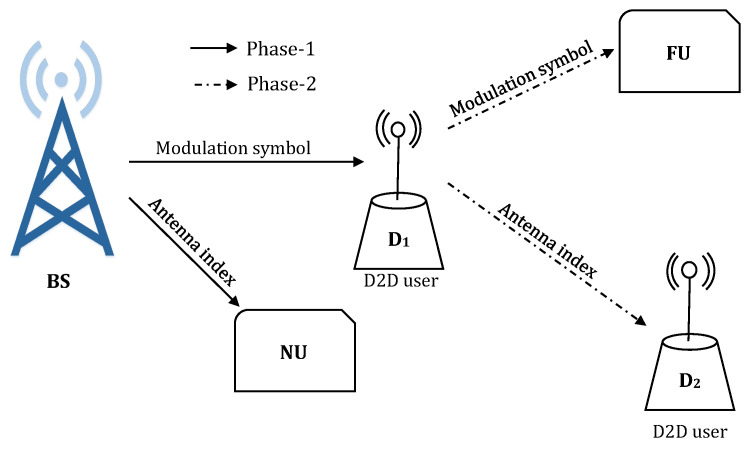
Proposed system model: S${}_{1}$.

**Figure 3:**
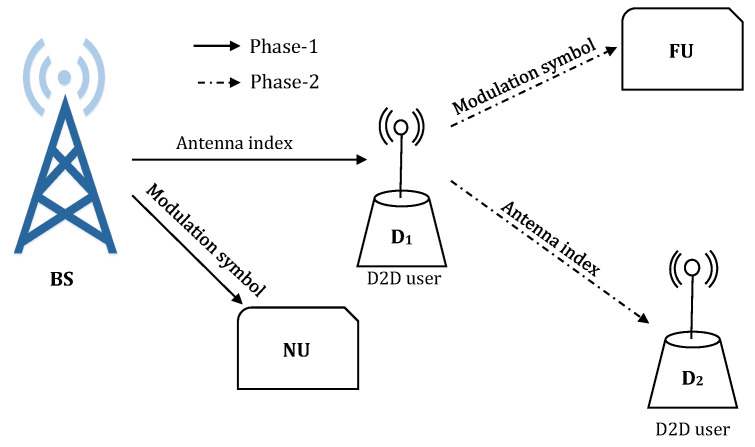
Proposed system model: S${}_{2}$.

Two different proposed network scenarios i.e., scenario-1 (S${}_{1}$) and scenario-2 (S${}_{2}$) in D2D-CRS-SM are shown in Figure 2 and Figure 3, respectively (Part of this work was presented in [51]. It should be mentioned that a very initial result was presented in  [51] by considering only the second scenario of the proposed network architecture in this paper. Moreover, the performance of [51] was studied in terms of BER and SE considering only BPSK and QPSK.). Each scenario consists of a BS, two cellular users (e.g., NU and FU) and a D2D transmitter-receiver pair (e.g., D${}_{1}$ and D${}_{2}$). The direct link between the BS and FU is assumed to be weak for data transmission because of poor conditions of channel. Hence, the direct link is not suitable for communication and it is neglected for this reason. So the BS solely depends upon D${}_{1}$ for the purpose of communication with FU, where D${}_{1}$ acts as half-duplex DF relay. The above assumptions are very much valid for cellular users such as FU situated far from BS e.g., a user located at the edge of a cellular network and suffers from very low SNR. Each node is a multi-antenna system. The communication nodes BS, NU, FU, D${}_{1}$ and D${}_{2}$ are equipped with A${}_{BS}$, A${}_{N}U$, A${}_{F}U$, A${}_{D_{1}}$, and A${}_{D_{2}}$ antennas, respectively. It is important to mention that the data transmission policy in S${}_{1}$ and S${}_{2}$ is identical except that antenna index of BS is the desired information of NU in S${}_{1}$, whereas modulation symbol of BS is the desired information of NU in S${}_{2}$.

For the channel between any two communication nodes *x* and *y*, it is assumed that the channel experiences independent Rayleigh flat fading plus additive white Gaussian noise. The channel coefficient is denoted by $h_{x,y}∼\mathrm{CN}\left(0,\lambda_{x,y}\right)$ with zero mean and variance $\lambda_{x,y}$, where $\left(x,y\right)\in \left\{BS,NU,FU,D_{1},D_{2}\right\}$ and x≠y. So the link gain will be $\alpha_{x,y}={\left|h_{x,y}\right|}^{2}$, where $\alpha_{x,y}$ is an exponentially distributed random variable with a scale parameter (or mean value) $\lambda_{x,y}\left(\lambda_{x,y}>0\right)$ [52,53]. The transmit powers of BS and D${}_{1}$ are represented as $P^{BS}$ and $P^{D_{1}}$, respectively. The average SNR is denoted by ρ. It is also assumed that the receivers are well aware of CSI i.e., CSI at NU and D${}_{1}$ during $P_{1}$ and CSI at FU and D${}_{2}$ during $P_{2}$. The data transmission process in the proposed D2D-CRS-SM is completed in two cooperative phases which are described in detail in the following sections.

### 2.1. Phase-1 ($P_{1}$): BS→ (D${}_{1}$, NU) Data Transmission

During $P_{1}$, the BS transmits to NU and D${}_{1}$ by invoking SM. The information bit stream of BS are mapped into two data carrying units: the $M_{1}$-PSK/QAM modulation order bits and the antenna index. In S${}_{1}$, the BS maps NU data bits as antenna indices and FU data bits as $M_{1}$-PSK/QAM modulation symbols. The mapping is shuffled in S${}_{2}$; NU data bits are mapped as $M_{1}$-PSK/QAM modulation symbols and FU data bits are mapped as antenna indices. Hence, in each transmission, the total number of bits that can be transmitted by BS in S${}_{1}$ and S${}_{2}$ are respectively given by [12,38]

(1) $$\begin{array}{cc} X_{1,S_{1}} & =Q_{1}+N_{1}\\ \; & =\log_{2}\left(A_{BS}\right)+\log_{2}\left(M_{1}\right), \end{array}$$

(2) $$\begin{array}{cc} X_{1,S_{2}} & =Q_{1}+N_{1}\\ \; & =\log_{2}\left(M_{1}\right)+\log_{2}\left(A_{BS}\right). \end{array}$$

where $Q_{1}$, $N_{1}$, and $M_{1}$ represent number of bits per NU symbol, number of bits per FU symbol, and modulation order used by BS, respectively. Various order of modulation can be used, e.g., BPSK, QPSK/4-QAM, 8-PSK/8-QAM, 16-PSK/16-QAM, 32-PSK/32-QAM and 64-PSK/64-QAM which represent corresponding values of $M_{1}$ = [2, 4, 8, 16, 32 and 64], respectively. Exploiting SM allows BS to activate only one of its antennas for a particular transmission. As a result, ICI is avoided and synchronization among the antennas is not required anymore. The data blocks transmitted by the BS is then de-mapped using iterative-maximum ratio combining (*i*-MRC) [12] technique by NU and D${}_{1}$ to retrieve their desired information.

From (1) and (2), it is apparent that there is a trade-off between the modulation order and the number of transmit antennas for any number of transmitted information bits. For example, three data bits per symbol can be sent with four transmit antennas using BPSK modulation. As a trade-off, the same three bits can be sent from two antennas using 4-QAM modulation.

### 2.2. Phase-2 ($P_{2}$): D${}_{1}$ → (D${}_{2}$, FU) Data Transmission

During $P_{2}$, D${}_{1}$ invokes SM as well to transmit data bits to FU and D${}_{2}$, only after decoding FU data bits from BS successfully. If the decoding of data bits is unsuccessful then D${}_{1}$ remains idle during $P_{2}$. Similar to $P_{1}$, the information bits to be transmitted by D${}_{1}$ is mapped into two data carrying units: the $M_{2}$-PSK/QAM modulation order bits and the antenna index. Here, the modulation order bits are the data bits received from BS that need to be forwarded to FU and the antenna index is associated with D${}_{1}$’s own information for transmitting to D${}_{2}$ as part of D2D communication. It should be mentioned that D${}_{1}$ must exploit $M_{2}=2^{N_{1}}$-PSK/QAM modulation, in order to retransmit the decoded data bits of FU as the number of bits in a symbol of FU is $N_{1}$. According to the concept of SM, only one antenna is allowed to be activated at D${}_{1}$ at a particular time instant. D${}_{1}$ selects the transmit antenna to be activated based on its own message symbol. Again, single antenna transmission avoids ICI along with synchronization among the antennas, as D${}_{1}$ exploits SM-MIMO to retransmit FU data and and its own data to D${}_{2}$. As a result, an interference free data reception is possible by both FU and D${}_{2}$. Now, the number of data bits that can be transmitted by D${}_{1}$ in S${}_{1}$ and S${}_{2}$ are respectively given by

(3) $$\begin{array}{cc} X_{2,S_{1}} & =N_{1}+N_{2}\\ \; & =\log_{2}\left(M_{1}\right)+\log_{2}\left(A_{D_{1}}\right), \end{array}$$

(4) $$\begin{array}{cc} X_{2,S_{2}} & =N_{1}+N_{2}\\ \; & =\log_{2}\left(A_{BS}\right)+\log_{2}\left(A_{D_{1}}\right). \end{array}$$

where $N_{2}$ is the number of bits in each symbol of D${}_{2}$. Then *i*-MRC is used by both D${}_{2}$ and FU, in order to de-map the data blocks from the received signal, where D${}_{2}$ recovers the antenna index and the FU extracts desired modulation symbol. The proposed D2D aided CRS with the transmit antenna selection technique used by BS and D${}_{1}$ is summarized in Algorithm 1. Moreover, the examples provided in Table 1 and Table 2 explain Algorithm 1 in a much simpler way. In Table 1, an example of SM mapping table at BS to transmit information to NU and FU is presented, whereas an example of SM mapping table at D${}_{1}$ is demonstrated in Table 2. Here, error free transmission is assumed for the examples provided in the tables to make the explanation of the algorithms simple.
**Algorithm 1** D2D-CRS-SM (A${}_{BS}$, $N_{1}$, $Q_{1}$ A${}_{D_{1}}$, $N_{2}$)1:**begin**2:U${}_{sm}$← block of data with length $Q_{1}$ corresponds to NU’s data3:F${}_{sm}$← block of data with length $N_{1}$ corresponds to FU’s data4:B${}_{sm}$← decoded block of data with length $N_{1}$ at D${}_{1}$ during $P_{1}$5:D${}_{sm}$←D${}_{1}$’s own data divided into block of length $N_{2}$⊳ Phase-I6:SM-based mapping table (e.g., Table 1) is constructed at BS.7:**for***i* = 0 to A${}_{BS}$ - 1 **do**8:    A${}_{index}=i_{b}$; equivalent binary bits of *i*9:    **if** U${}_{sm}$ = A${}_{index}$ [S${}_{1}$] or, F${}_{sm}$ = A${}_{index}$ [S${}_{2}$] **then**10:        BS transmits F${}_{sm}$ in S${}_{1}$ and U${}_{sm}$ in S${}_{2}$ by $i_{th}$ antenna11:    **end if**12:**end for**13:NU and D${}_{1}$ attempts to decode their corresponding information using *i*-MRC.⊳ Phase-II14:**if** decoding is successful **then**15:    **for** *i* = 0 to $A_{D_{1}}$ - 1 **do**16:        A${}_{index}=i_{b}$; equivalent binary bits of *i*17:        **if** D${}_{sm}$ = A${}_{index}$ **then**18:           D${}_{1}$ transmits B${}_{sm}$ by *i*th antenna19:        **end if**20:    **end for**21:**else**22:    D${}_{1}$ remains idle during $P_{2}$.23:**end if**24:**end**

## 3. Performance Analysis

In this section, we analyze the performance of D2D-CRS-SM, in terms of BER and SE over independent Rayleigh fading channels.

### 3.1. Bit-Error-Rate Computation

Data transmission from BS to FU is a dual-hop transmission. As mentioned in the previous section, D${}_{1}$ forwards the signal of FU during $P_{2}$, only if the received signal from BS is correctly estimated during $P_{1}$, otherwise D${}_{1}$ remains silent during $P_{2}$. Hence, to determine the BER at FU, we have to consider the estimated error of links BS→ D${}_{1}$ and D${}_{1}$→FU during $P_{1}$ and $P_{2}$, respectively. Let $P_{e,FU}^{S,P_{1}}$ and $P_{e,FU}^{A,P_{1}}$ denote the probability of incorrect estimation of transmitted data of FU at D${}_{1}$ in S${}_{1}$ and S${}_{2}$, respectively during $P_{1}$. Again, let $P_{e,FU}^{S,P_{2}}$ denote the probability of incorrect estimation of transmitted data at FU during $P_{2}$. Therefore, the end-to-end (e2e) probability of error at FU in S${}_{1}$ and S${}_{2}$ can be written respectively as

(5) $$\begin{array}{cc} P_{e2e,FU}^{S_{1}}= & 1-\left(1-P_{e,FU}^{S,P_{1}}\right)\left(1-P_{e,FU}^{S,P_{2}}\right)\\ = & P_{e,FU}^{S,P_{1}}+P_{e,FU}^{S,P_{2}}-P_{e,FU}^{S,P_{1}}\times P_{e,FU}^{S,P_{2}}, \end{array}$$

(6) $$\begin{array}{cc} P_{e2e,FU}^{S_{2}}= & 1-\left(1-P_{e,FU}^{A,P_{1}}\right)\left(1-P_{e,FU}^{S,P_{2}}\right)\\ = & P_{e,FU}^{A,P_{1}}+P_{e,FU}^{S,P_{2}}-P_{e,FU}^{A,P_{1}}\times P_{e,FU}^{S,P_{2}}. \end{array}$$

Apart from the node FU, other communication nodes (e.g., NU and D${}_{2}$) in D2D-CRS-SM receive their data bits through the direct link. Hence, during $P_{1}$, the probability of incorrect estimation of transmitted data at NU in S${}_{1}$ and S${}_{2}$ are denoted by $P_{e,NU}^{A,P_{1}}$ and $P_{e,NU}^{S,P_{1}}$, respectively. In addition, the probability of incorrect estimation of antenna index at D${}_{2}$ is denoted by $P_{e,D_{2}}^{A,P_{2}}$ during $P_{2}$. It is worth noting the following observations:

- NU is only concerned about the correct estimate of antenna index and modulation symbol during $P_{1}$ in S${}_{1}$ and S${}_{2}$, respectively. So the overall error probability of NU is only affected by the erroneous estimation of antenna index and modulation symbol in S${}_{1}$ and S${}_{2}$, respectively.
- The desired information of FU is the opposite of NU in $P_{1}$, whereas the desired information of FU in $P_{2}$ is the transmitted modulation symbol by D${}_{1}$ irrespective of S${}_{1}$ and S${}_{2}$. Therefore, the overall error probability of FU as shown in (5) and (6) is only affected by erroneous estimation of modulation symbol and antenna index in S${}_{1}$ and S${}_{2}$, respectively during $P_{1}$ as well as erroneous estimation of modulation symbol during $P_{2}$.
- Lastly, the overall error probability of D2D user D${}_{2}$ is only affected by the erroneous estimation of antenna index in $P_{2}$ only.

Recall that BS and D${}_{1}$ transmit by activating only one antenna due to the exploitation of SM. Hence, as a special case, any transmitted *M*-PSK/*M*-QAM symbol estimation at D${}_{1}$ and FU in S${}_{1}$ can be considered as $1\times A_{D_{1}}$ and $1\times A_{FU}$ MRC detection, respectively [12]. Similarly, any transmitted *M*-PSK/*M*-QAM symbol estimation at NU in S${}_{2}$ can be considered as $1\times A_{NU}$ MRC detection. The analytical formulation of error probabilities of FU and NU under this special case is provided in Appendix A. Moreover, the overall error probability of the corresponding user for erroneous estimation of antenna number can be calculated by [12] (Equation (10)).

### 3.2. Spectral Efficiency Computation

#### 3.2.1. Spectral Efficiency of FU

Recall that FU is not directly connected to the BS. It needs the assistance of D${}_{1}$ to communicate with the BS. Therefore, both the first hop (BS→ D${}_{1}$) and second hop (D${}_{1}$→ FU) links need to be considered to compute the SE of FU. Now, using [54] (Equation (22)) and (5), the e2e overall SE of the FU in S${}_{1}$ is obtained as

(7) $$\begin{array}{cc} R_{SE,FU}^{S_{1}}= & \frac{1}{2}N_{1}\{1+P_{e2e,FU}^{S_{1}}\log_{2}\left(P_{e2e,FU}^{S_{1}}\right)\\ \; & +\left(1-P_{e2e,FU}^{S_{1}}\right)\log_{2}\left(1-P_{e2e,FU}^{S_{1}}\right)\}. \end{array}$$

where $N_{1}=\log_{2}\left(M_{1}\right)$ in S${}_{1}$ and $N_{1}=\log_{2}\left(A_{BS}\right)$ in S${}_{2}$.

Again, using [54] (Equation (22)) and (6), the e2e overall SE of the FU in S${}_{2}$ is obtained as

(8) $$\begin{array}{cc} R_{SE,FU}^{S_{2}}= & \frac{1}{2}N_{1}\{1+P_{e2e,FU}^{S_{2}}\log_{2}\left(P_{e2e,FU}^{S_{2}}\right)\\ \; & +\left(1-P_{e2e,FU}^{S_{2}}\right)\log_{2}\left(1-P_{e2e,FU}^{S_{2}}\right)\}. \end{array}$$

Please note that the data transmission policy for S${}_{1}$ and S${}_{2}$ is different in $P_{1}$ but identical in $P_{2}$. In (7) and (8), the scaling factor $\frac{1}{2}$ is used to denote that the e2e data transmission is divided into two cooperative phases and each phase requires one time slot.

#### 3.2.2. Spectral Efficiency of NU

As discussed in Section 2, NU directly communicates with the BS via the direct link in $P_{1}$. However, NU keeps silent in $P_{2}$. The overall SE of NU in S${}_{1}$ and S${}_{2}$ is therefore obtained respectively as

(9) $$\begin{array}{cc} R_{SE,NU}^{S_{1}}= & \frac{1}{2}Q_{1}\{1+P_{e,NU}^{A,P_{1}}\log_{2}\left(P_{e,NU}^{A,P_{1}}\right)\\ \; & +\left(1-P_{e,NU}^{A,P_{1}}\right)\log_{2}\left(1-P_{e,NU}^{A,P_{1}}\right))\}, \end{array}$$

(10) $$\begin{array}{cc} R_{SE,NU}^{S_{2}}= & \frac{1}{2}Q_{1}\{1+P_{e,NU}^{S,P_{1}}\log_{2}\left(P_{e,NU}^{S,P_{1}}\right)\\ \; & +\left(1-P_{e,NU}^{S,P_{1}}\right)\log_{2}\left(1-P_{e,NU}^{S,P_{1}}\right))\}. \end{array}$$

where $Q_{1}=\log_{2}\left(A_{BS}\right)$ in S${}_{1}$ and $Q_{1}=\log_{2}\left(M_{1}\right)$ in S${}_{2}$. In (9) and (10), the scaling factor $\frac{1}{2}$ is used to denote that NU is unable to receive data from BS in $P_{2}$.

#### 3.2.3. Spectral Efficiency of D2D Link D${}_{1}$ → D${}_{2}$

Recall that D${}_{1}$ stays silent during $P_{1}$ but forwards the decoded symbol of FU during $P_{2}$. Irrespective of S${}_{1}$ and S${}_{2}$, the desired information of D2D receiver D${}_{2}$ is the antenna index of D${}_{1}$. Therefore, the overall SE of the D2D link D${}_{1}$ → D${}_{2}$ is expressed as

(11) $$\begin{array}{cc} R_{SE,D_{2}}= & \frac{1}{2}N_{2}\{1+P_{e,D_{2}}^{A,P_{2}}\log_{2}\left(P_{e,D_{2}}^{A,P_{2}}\right)\\ \; & +\left(1-P_{e,D_{2}}^{A,P_{2}}\right)\log_{2}\left(1-P_{e,D_{2}}^{A,P_{2}}\right))\}. \end{array}$$

In (11), the scaling factor $\frac{1}{2}$ is used to denote that D${}_{2}$ is unable to receive data from D${}_{1}$ in $P_{1}$.

#### 3.2.4. Sum Spectral Efficiency of the Proposed D2D-CRS-SM

Using (7), (9), and (11), the sum SE of the proposed D2D-CRS-SM in S${}_{1}$ is obtained as

(12) $$\begin{array}{cc} R_{SE,sum}^{S_{1}}= & R_{SE,FU}^{S_{1}}+R_{SE,NU}^{S_{1}}+R_{SE,D_{2}}. \end{array}$$

Moreover, using (8), (10), and (11), the sum SE of the proposed D2D-CRS-SM in S${}_{2}$ is obtained as

(13) $$\begin{array}{cc} R_{SE,sum}^{S_{2}}= & R_{SE,FU}^{S_{2}}+R_{SE,NU}^{S_{2}}+R_{SE,D_{2}}. \end{array}$$

## 4. Numerical Results and Discussions

This section presents the performance of the proposed D2D-CRS-SM, in terms of BER, SE, and sum SE over independent and identically distributed Rayleigh fading channel. The channel coefficient of each link is assumed to be of zero mean and unit variance. Moreover, unless otherwise stated, the simulation parameters are summarized in Table 3.

### 4.1. Bit-Error-Rate

#### 4.1.1. Bit-Error-Rate in S${}_{1}$

**Figure 4:**
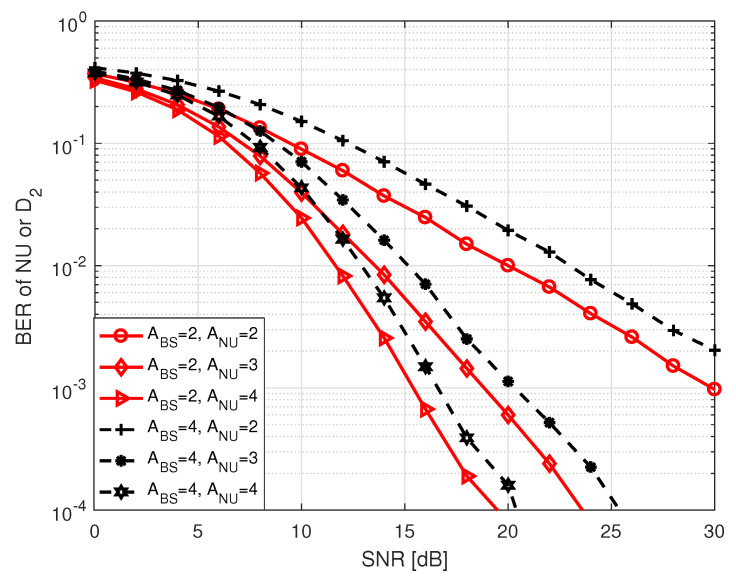
BER of NU in S${}_{1}$ or BER of D${}_{1}$–D${}_{2}$ pair in both S${}_{1}$ and S${}_{2}$. For D${}_{1}$–D${}_{2}$ pair, we set A${}_{D_{1}}$ = A${}_{B}S$ and A${}_{D_{2}}$ = A${}_{N}U$.

In Figure 4, we present BER performance of both NU and D${}_{2}$ with respect to SNR, where SNR varies from 0 to 30 dB. In S${}_{1}$, NU and D${}_{2}$ have identical BERs because their BERs depend only on the incorrect antenna estimation. This is also true for BER of D${}_{2}$ in S${}_{2}$. The terms in legend of Figure 4 represents the number of transmit and receive antennas. We observed that BER decreases with increasing SNR values, as expected. We also observed that the BER performance improves with an increase in the number of receive antennas. This is due to the increase of diversity order with an increase of the receive antenna numbers. On the other hand, an increase in the number of transmit antennas triggers more error. The reason is that when the number of transmit antennas increases, the likelihood of erroneous detection of antennas increases [12].

**Figure 5:**
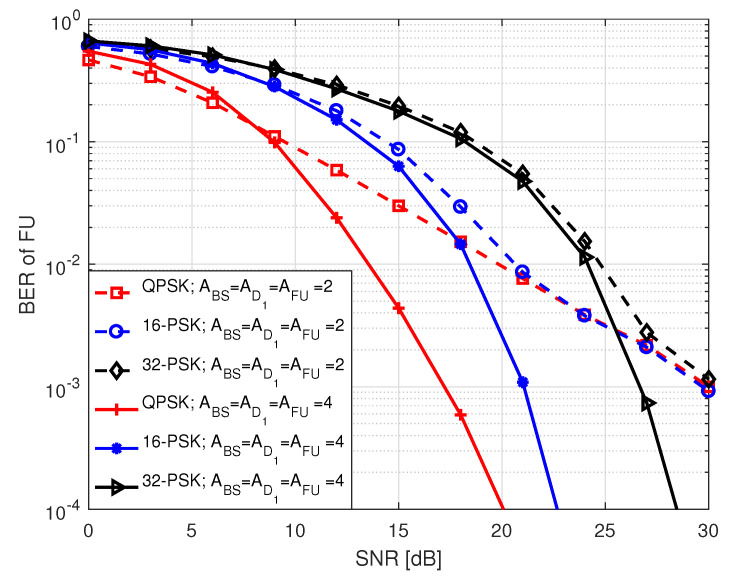
BER of FU in S${}_{1}$ for *M*-PSK.

**Figure 6:**
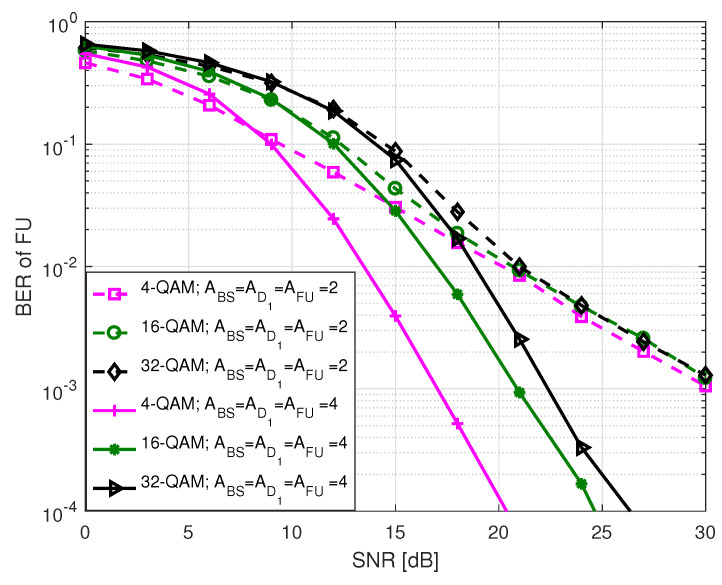
BER of FU in S${}_{1}$ for *M*-QAM.

Figure 5 and Figure 6 illustrate the BER performance of FU in S${}_{1}$ for different order of PSK and QAM modulation as a function of SNR, respectively, where two different types of antenna configurations are considered. Here, SNR ranges between 0 and 30 dB. The term in legends of the two figures denotes the number of antennas at BS (A${}_{BS}$), D${}_{1}$ (A${}_{D_{1}}$), FU (A${}_{FU}$), and *M*-PSK/QAM modulation used by BS and D${}_{1}$ to transmit FU data bits. In each instance, BER declines with an increase in SNR which is expected. We noted that in both figures higher order of modulation has higher BER compared to lower order of modulation when BS, D${}_{1}$ and FU are all equipped with 4 antennas. This is because of the fact that when the number of modulation order increases, the likelihood of erroneous detection of constellation points increases due to the reduction of Euclidean distances among the points. Similar trend is observed in BER where all nodes are equipped with 2 antennas except that the BER curves tend to get closer to each other at high SNR.

**Figure 7:**
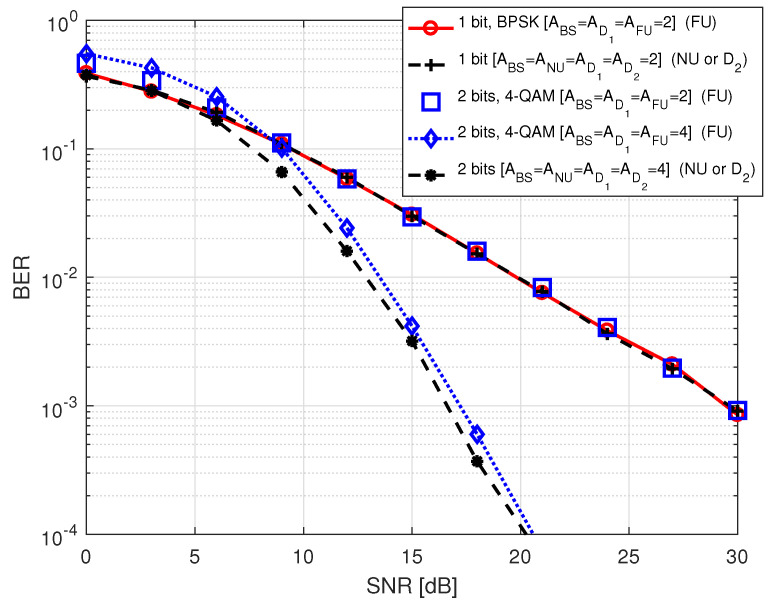
BER comparison among the users in S${}_{1}$.

In Figure 7, we plot BER of the different users in S${}_{1}$ against SNR for a comparative analysis, where SNR varies between 0 and 30 dB. The legend terms show the corresponding information as we mentioned for the previous figures and additionally includes the transmitted number of data bits. Considering the same order of modulation, it is noticed that FU gives better BER performance with 4 antennas at all involving nodes compared to 2 antennas at all involving nodes. We also noticed similar trend in the BER performance of NU or D${}_{2}$, i.e., BER shows improved performance when all involving nodes are equipped with 4 antennas. It is worth noting that BER of NU or D${}_{2}$ outperforms BER of FU for the transmission of the same number of data bits (e.g., 2 bits) when all the nodes in the system are equipped with four antennas. Recall that antenna indices are the desired information of NU and D${}_{2}$ and modulation symbol is the desired information of FU in S${}_{1}$.

#### 4.1.2. Bit-Error-Rate in S${}_{2}$

**Figure 8:**
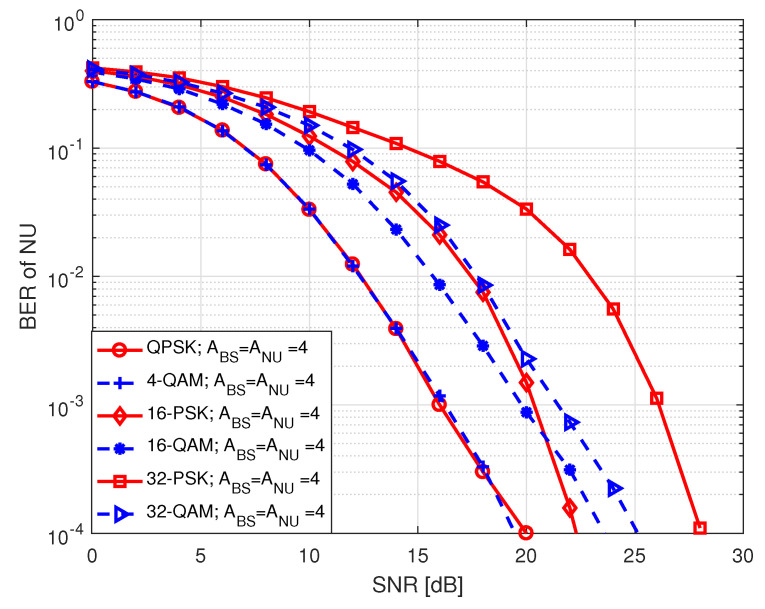
BER of NU in S${}_{2}$.

Figure 8 demonstrates BER performance of NU in S${}_{2}$ as a function of SNR for different order of PSK and QAM modulation, considering the BS and NU are equipped with the same number of of antennas. Here, SNR ranges between 0 to 30 dB. In each instance, BER gradually declines with increasing SNR which is expected. We observed that for both PSK and QAM higher order of modulation corresponds to higher BER. As we mentioned before, this is because of the fact that when the number of modulation order increases, the likelihood of erroneous detection of constellation points increases. We also noticed that for higher order of modulation BER performance with QAM is better compared to the ones with PSK.

**Figure 9:**
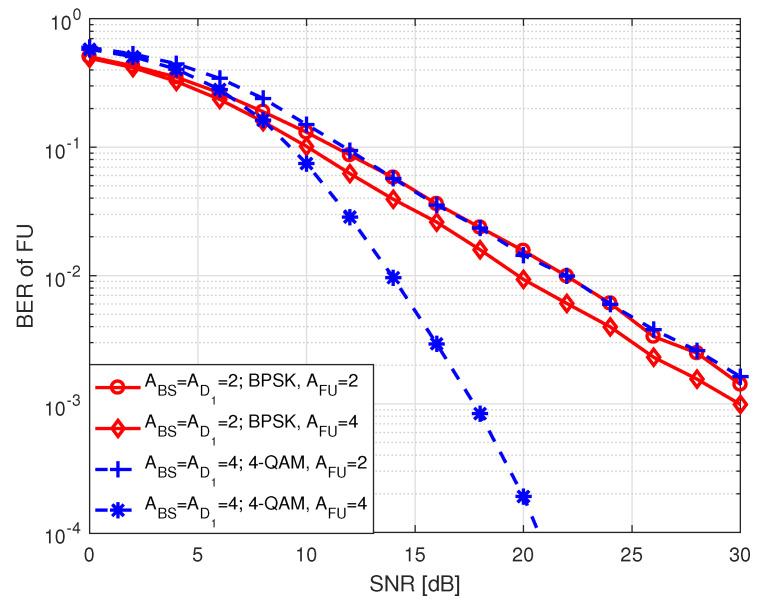
BER of FU in S${}_{2}$.

In Figure 9, we portrait e2e BER performance of FU in S${}_{2}$ with respect to SNR, where SNR holds values from 0 to 30 dB. The term in inset represents the following parameters: (i) A${}_{BS}$, A${}_{D_{1}}$, and A${}_{FU}$ denote the number of antennas at BS, D${}_{1}$ and FU, respectively, and (ii) D${}_{1}$ exploits SM to forward de-mapped FU information with $2^{N_{1}}$-PSK/QAM modulation. It is noted that an increase in the number of antennas at FU improves the BER performance, considering the same number of antennas at BS and D${}_{1}$. When the number of antennas at a receiver increases, the diversity order increases, which in turn improves the BER of FU with an increase in the number of receiving antennas. It is also observed that the improvement in BER is dramatic when the BS and D${}_{1}$ are equipped with higher number of antennas.

**Figure 10:**
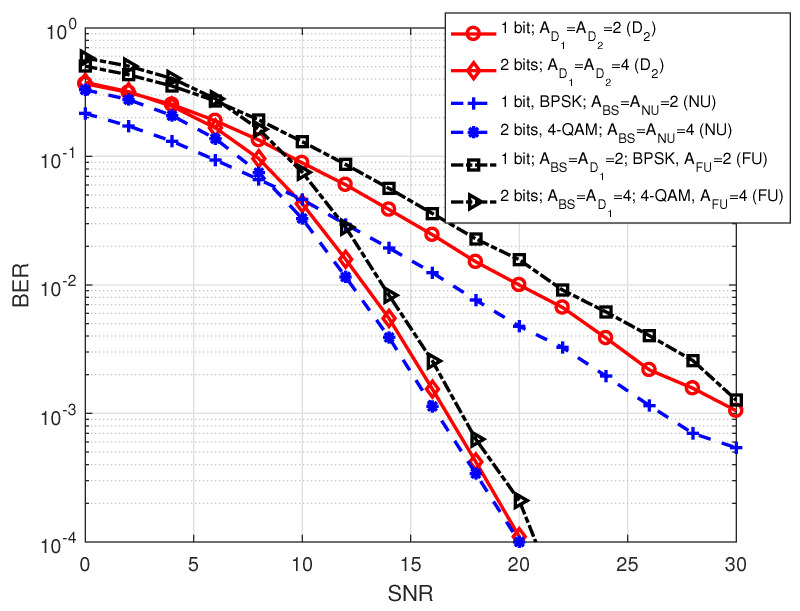
BER comparison among the users in S${}_{2}$.

In Figure 10, we show BER of the different users in S${}_{2}$ against SNR for a comparative analysis. The legend terms denote the corresponding information as we mentioned for the previous two figures and additionally includes the transmitted number of data bits. Considering similar antenna configurations at the nodes involved in transmission, it is noticed that BER of NU is lower than other two users (D${}_{2}$ and FU) while BER of FU is higher than others for the transmission of same number of data bits.

### 4.2. Spectral Efficiency

#### 4.2.1. Spectral Efficiency in S${}_{1}$

**Figure 11:**
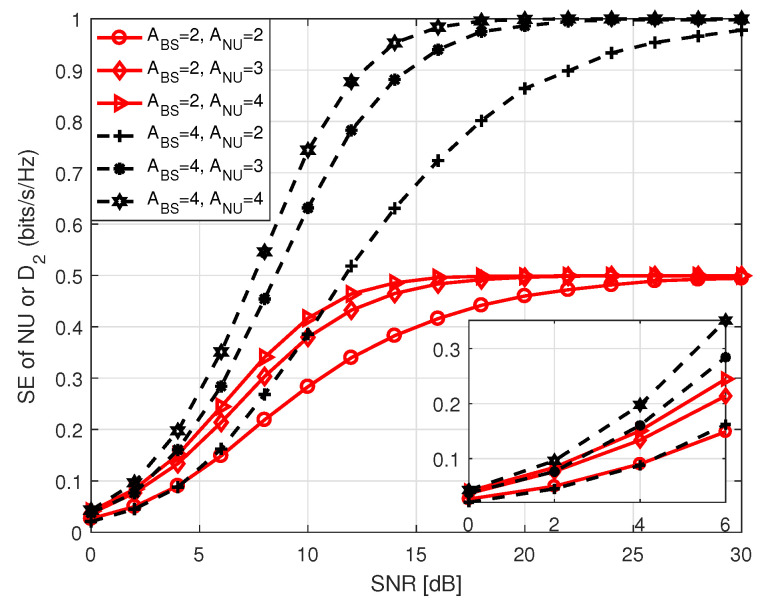
SE of NU in S${}_{1}$ or SE of D${}_{1}$–D${}_{2}$ pair in both S${}_{1}$ and S${}_{2}$. For D${}_{1}$–D${}_{2}$ pair, we set A${}_{D_{1}}$ = A${}_{B}S$ and A${}_{D_{2}}$ = A${}_{N}U$.

**Figure 12:**
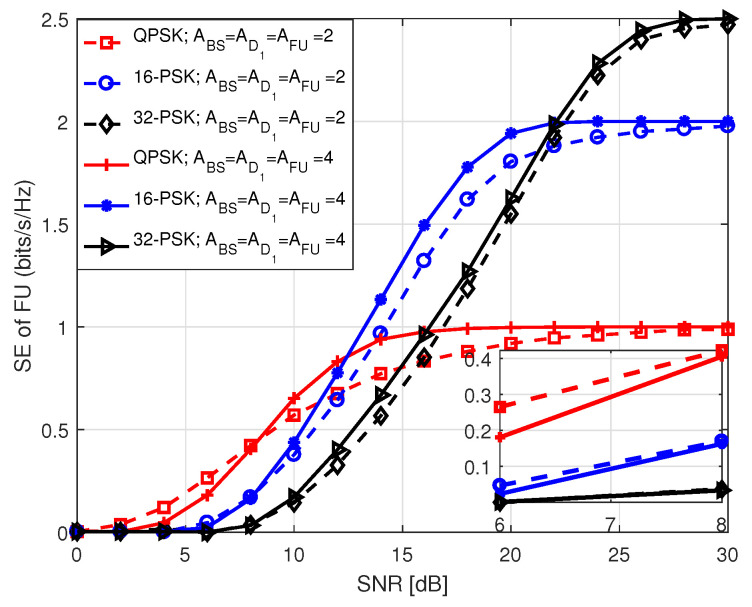
SE of FU in S${}_{1}$ for *M*-PSK.

**Figure 13:**
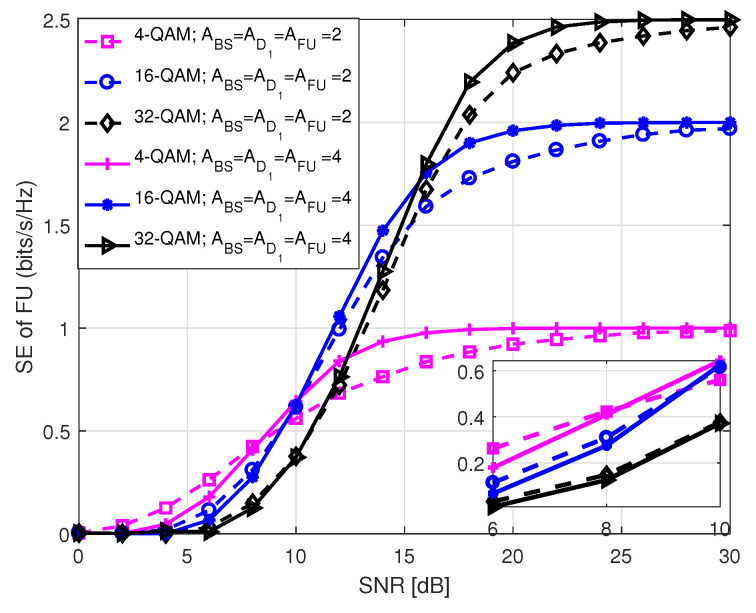
SE of FU in S${}_{1}$ for *M*-QAM.

Figure 11 presents SE of both NU and D${}_{2}$ in S${}_{1}$ with respect to SNR that holds values between 0 and 30 dB. As we mentioned in the system model, SE of NU and D${}_{2}$ solely depends on the antenna indices. Hence, their SE performance is identical in S${}_{1}$ for the same antenna configuration. On the contrary, the SE performance of FU depends on the modulation order employed by BS and D${}_{1}$, during $P_{1}$ and $P_{2}$, respectively in S${}_{1}$. It is observed that SE curves approach to the theoretical number of data bits at high SNR. This is because of the fact that at high SNR, BER tends to zero causing SE to reach its theoretical upper limit. It is also identified that an increase in the number of receiving antennas improves SE performance with the same number of transmitting antennas because of the improvement in diversity order. In Figure 12 and Figure 13, we illustrate SE performance of FU in S${}_{1}$ for different PSK and QAM modulation order against SNR, considering two distinct antenna configurations. Here, SNR holds values from 0 to 30 dB. The term in legends of the figures represents the parameters as mentioned earlier in Figure 5 and Figure 6. The SE curves in both figures approach to the theoretical number of bits with increasing SNR, as expected. It is noticed that for the same antenna configuration SE performance is better with lower order of modulation compared to the ones with higher order of modulation at low SNR. However, the system shows better SE performance with higher modulation order for medium to high SNR.

**Figure 14:**
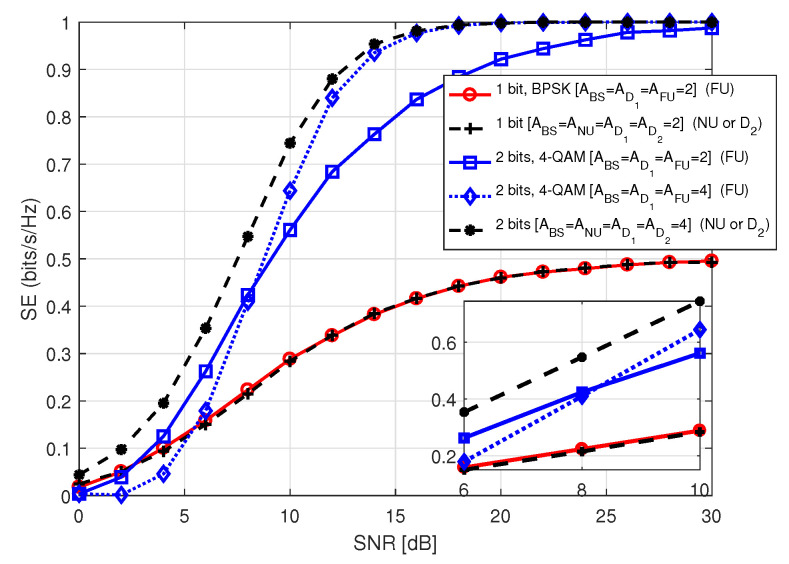
SE comparison among the users in S${}_{1}$.

Figure 14 demonstrates a comparative analysis of SE performance among different users in S${}_{1}$. The legend terms denote the corresponding parameters as mentioned earlier and additionally includes the transmitted number of data bits. As seen in the previous three figures, the SE curves here also lead to theoretical number of data bits eventually. We observe that SE performance of NU or D${}_{2}$ is better than that of FU for the transmission of same number of data bits with a higher number of antennas at all of the involving nodes. We also observed that SE performance of FU with a higher number of antennas at all the nodes involved in transmission is lower compared to the one with a lower number of antennas at low SNR. On the other hand, for a higher number of antennas case, SE is better for medium to high SNR.

#### 4.2.2. Spectral Efficiency in S${}_{2}$

**Figure 15:**
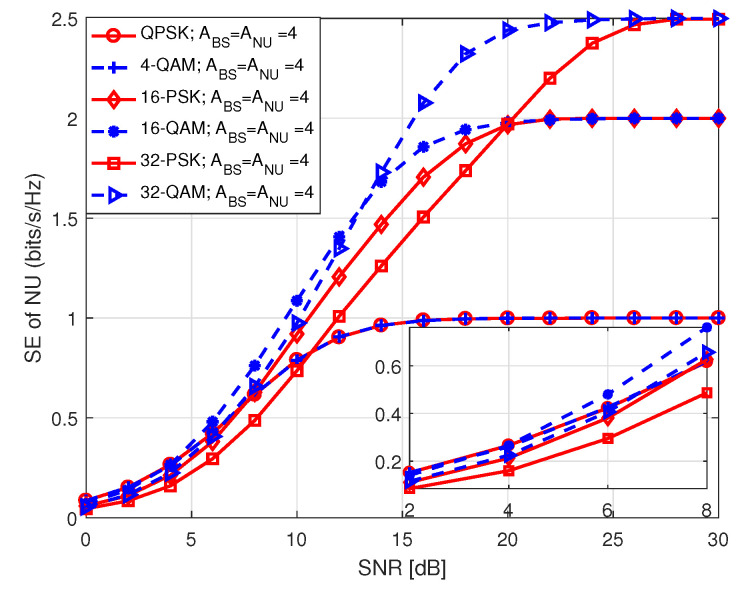
SE of NU in S${}_{2}$.

In Figure 15, we portrait SE performance of NU in S${}_{2}$ for *M*-PSK/QAM modulation with respect to SNR, where SNR holds values between 0 and 30 dB. The term in legend of the figure denote *M*-PSK/QAM modulation order used by BS to transmit data to NU, the number of transmit antennas at BS (A${}_{BS}$), and the number of receive antennas at NU (A${}_{NU}$). It is noticed that the system achieves lower SE employing higher order of modulation at low SNR compared to the ones using lower order of modulation with identical antenna configuration at BS and NU. We noted that SE performance with QAM modulation is better than that with PSK modulation for the same modulation order. However, in each case, the SE curves approach the theoretical number of data bits with increasing SNR because of the same reason as mentioned before.

**Figure 16:**
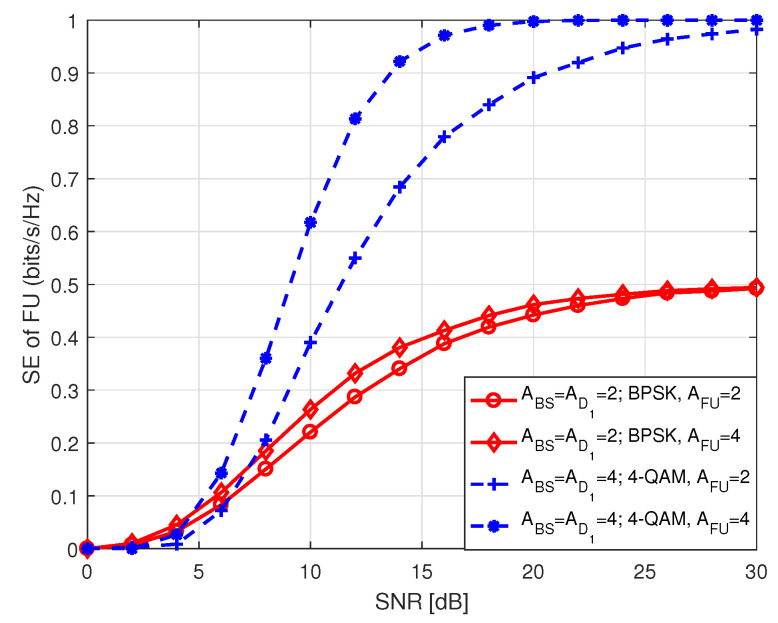
SE of FU in S${}_{2}$.

Figure 16 depicts SE performance of FU in S${}_{2}$ with respect to SNR, where SNR ranges between 0 and 30 dB. The term in legend represents the parameters as mentioned earlier in Figure 9. Here, the SE curves lead to theoretical number of data bits with increasing SNR which is expected. It is duly noted that an increase in the number of antennas at FU improves SE performance of the system with the same antenna configuration at BS and D${}_{1}$, and same order of modulation used by D${}_{1}$.

**Figure 17:**
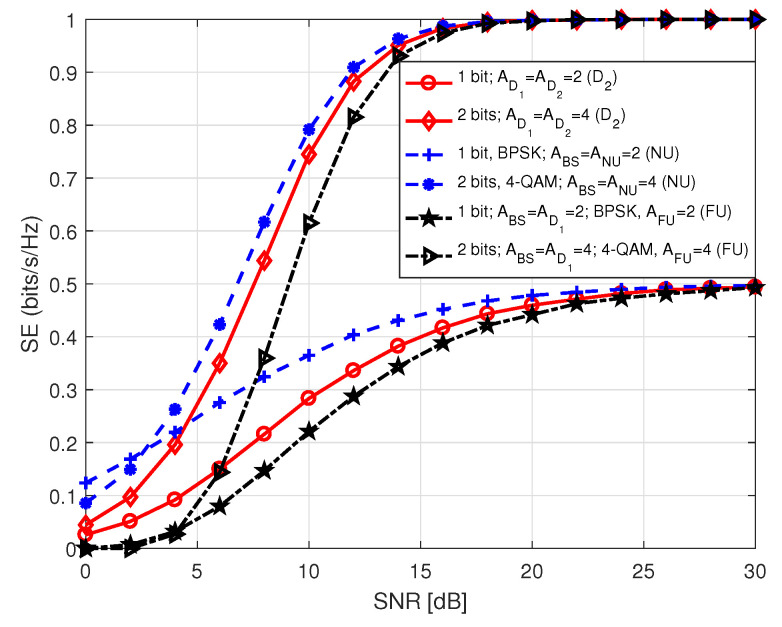
SE comparison among the users in S${}_{2}$.

In Figure 17, we plot SE of different users in S${}_{2}$ against SNR for a comparative analysis. The legend terms show the corresponding parameters as mentioned earlier and additionally includes the transmitted number of data bits. As seen in the previous two figures, the SE curves here also lead to theoretical number of data bits eventually. We observed that NU achieves higher SE performance than other two users (D${}_{1}$ and FU) while SE of FU is lower than others for the transmission of equal number of data bits with a similar antenna configuration at the nodes involved in transmission.

#### 4.2.3. Sum Spectral Efficiency

**Figure 18:**
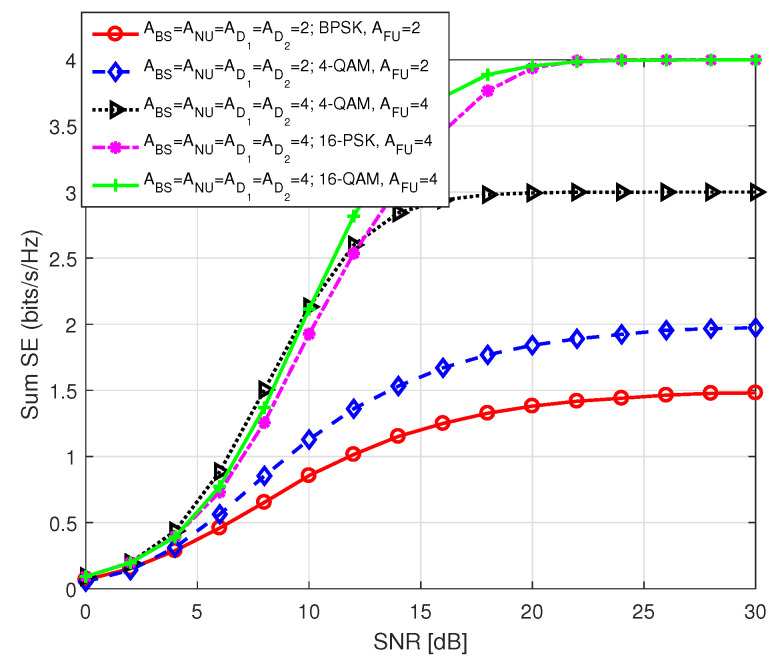
Sum SE in S${}_{1}$ for different combinations of antenna number and modulation order.

Figure 18 portraits sum SE of the users in S${}_{1}$ against SNR for different antenna combinations and order of modulations. The inset term represents the following parameters: (i) the number of antennas at BS (A${}_{BS}$), NU (A${}_{NU}$), D${}_{1}$ (A${}_{D_{1}}$), D${}_{2}$ (A${}_{D_{2}}$), and FU (A${}_{FU}$), respectively, and (ii) the modulation order used by BS and D${}_{1}$ to transmit FU symbol. We considered same number of antennas at all users for each instance to make consistent transmission. Please note that both BS and D${}_{1}$ should use same modulation order for efficient transmission of information from BS to FU via D${}_{1}$, according to our system model; otherwise, inconsistency in data transmission will arise between $P_{1}$ and $P_{2}$. We noted that in each instance sum SE curves approach to theoretical number of data bits which is anticipated because of the same reason as mentioned before. We recall that in S${}_{1}$, FU data bits depend on the modulation order employed by BS and D${}_{1}$. We observed that a higher order of modulation can be used to transmit FU information at a higher rate. This correspondingly effects the total SE of S${}_{1}$. As QAM is more spectral efficient than PSK modulation, we noticed that sum SE with QAM is better than that with PSK for the same order of modulation with identical antenna combination.

**Figure 19:**
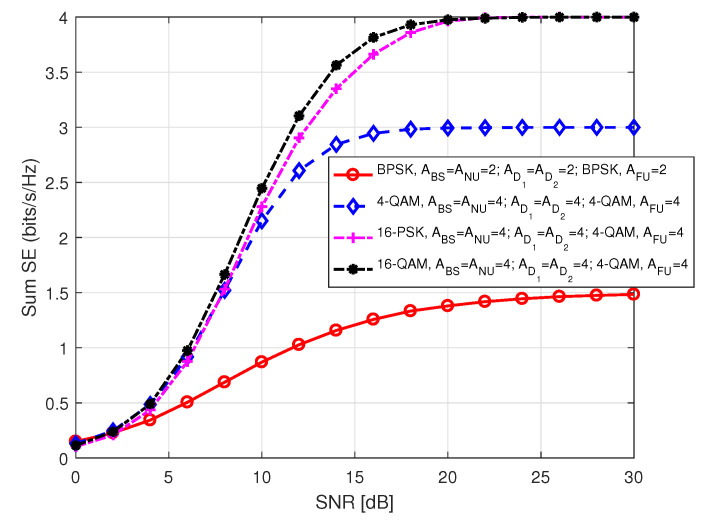
Sum SE in S${}_{2}$ for different combinations of antenna number and modulation order.

Figure 19 depicts sum SE of the users in S${}_{2}$ against SNR for different antenna combinations and order of modulations. The inset term represents the following parameters: (i) BS uses M-PSK/QAM modulation to transmit NU information, (ii) the number of antennas at BS (A${}_{BS}$), NU (A${}_{NU}$), D${}_{1}$ (A${}_{D_{1}}$), D${}_{2}$ (A${}_{D_{2}}$), and FU (A${}_{FU}$), respectively, and (iii) the modulation order used by D${}_{1}$ to transmit FU data symbol. We considered same number of antennas at all users for each instance to make consistent transmission. We noted that in each instance sum SE curves approach to theoretical number of data bits which is obvious. Recall that in S${}_{2}$, NU data bits depend on the modulation order used by BS, whereas FU data bits depend on the number of antennas at BS. We observed that a higher order of modulation can be used by BS to transmit NU information at a higher rate, whereas D${}_{1}$ is limited to use a modulation order that corresponds to the number of antennas available at BS to forward FU information. Different modulation orders and antenna combinations effect the total SE correspondingly.

**Figure 20:**
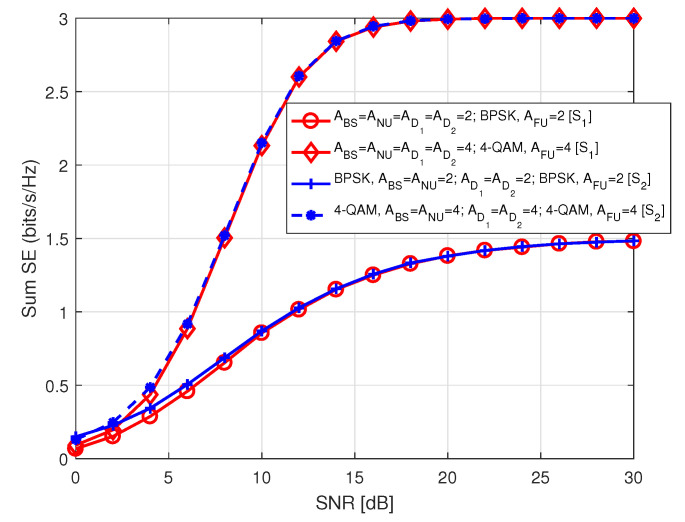
Sum SE comparison between S${}_{1}$ and S${}_{2}$.

Finally, we show a comparison of sum SE between our proposed scenarios S${}_{1}$ and S${}_{2}$ in Figure 20. We observed that sum of SE in S${}_{1}$ and S${}_{2}$ are quite identical to each other for the same combinations of antenna and modulation order. However, it needs to be mentioned that FU has the privilege of higher data rate through adoption of higher order of modulation in S${}_{1}$, whereas in S${}_{2}$, NU avails the benefits of modulation order.

### 4.3. Comparison between the Proposed D2D-CRS-SM and VBLAST MIMO with ZF Detection

**Figure 21:**
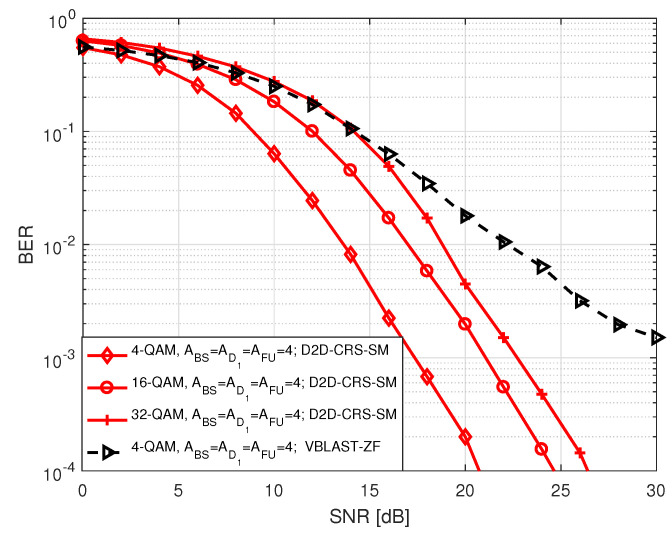
A comparative study between the proposed D2D-CRS-SM and VBLAST-ZF in terms of BER of FU in S${}_{1}$.

**Figure 22:**
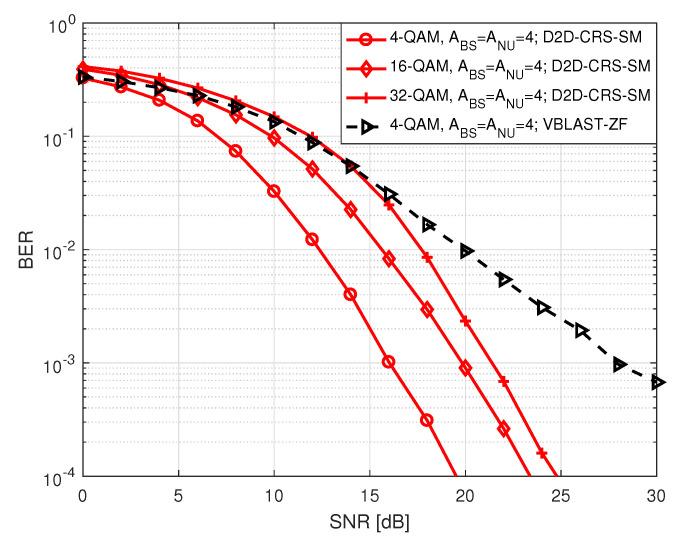
A comparative study between the proposed D2D-CRS-SM and VBLAST-ZF in terms of BER of NU in S${}_{2}$.

Comparative results between the D2D-CRS-SM and spatial multiplexing MIMO (e.g., VBLAST) with ZF Detection, in terms of BER and SE are presented here. Recall that modulation order (i.e., symbol information) is the desired information of FU and NU in S${}_{1}$ and S${}_{2}$, respectively. On the contrary, antenna indices of BS is the desired information of NU and FU in S${}_{1}$ and S${}_{2}$, respectively. Therefore, for a fair comparison between the proposed D2D-CRS-SM and VBLAST-ZF, only the incorrect estimation of symbol information of FU in S${}_{1}$ and NU in S${}_{2}$ is considered in Figure 21 and Figure 22, respectively. From both figures, it is apparent that for the same set of modulation order and antenna configuration, the proposed SM-based model (i.e., D2D-CRS-SM) outperforms the VBLAST-ZF for any value of SNR, although higher number of bits can be transmitted by VBLAST-ZF than D2D-CRS-SM. Please note that the SE loss is not limitation of our system. Rather, it is the limitation of SM-based system [12,55]. Furthermore, it should be mentioned that SE obtained by SM-based system is sub-optimal because only one antenna of transmitter is activated at a particular time instant to transmit information. As a result, SM-based system can achieve lower SE than spatial multiplexing MIMO technique (e.g., VBLAST). Although SM-based systems provide sub-optimal SE, it has many advantages including simpler transmitter design, reduced receiver complexity, lower transmit power supply and so on. However, there are two alternative ways for SM-based system to achieve the SE as capacity achieving VBLAST: (i) having the same modulation order in both systems, SM-based systems should use a larger number of transmit antennas, or (ii) having the same antenna configuration in both systems, SM-based systems should use a higher modulation order [55]. In Table 4, the maximum achievable theoretical sum SE comparison is summarized between the proposed D2D-CRS-SM and VBLAST-ZF. From the table, it is apparent that the proposed system can achieve a competitive sum SE as in VBLAST-ZF by increasing modulation order for the same antenna configuration. Moreover, as shown in the simulation figures of the proposed system, by keeping the same modulation order, exploiting a larger number of transmit antennas can also achieve a better SE.

The maximum achievable theoretical sum SE of D2D-CRS-SM in S${}_{1}$ and S${}_{2}$ is given respectively by

(14) $$\begin{array}{cc} R_{SE,sum,S_{1}}^{D2D-CRS-SM}= & \frac{1}{2}\left\{\underset{NU}{\underline{\log_{2}A_{B}S}}+\underset{FU}{\underline{\min(\log_{2}M_{1},\log_{2}M_{2})}}+\underset{D_{2}}{\underline{\log_{2}A_{D_{1}}}}\right\}, \end{array}$$

(15) $$\begin{array}{cc} R_{SE,sum,S_{2}}^{D2D-CRS-SM}= & \frac{1}{2}\left\{\underset{NU}{\underline{\log_{2}M_{1}}}+\underset{FU}{\underline{\min(\log_{2}A_{B}S,\log_{2}M_{2})}}+\underset{D_{2}}{\underline{\log_{2}A_{D_{1}}}}\right\}. \end{array}$$

On the contrary, maximum achievable theoretical sum SE of VBLAST-ZF is given by

(16) $$\begin{array}{cc} R_{SE,sum}^{VBLAST}= & \frac{1}{4}\left\{\underset{NU}{\underline{\left(A_{B}S\times N_{1}\right)}}+\underset{FU}{\underline{\min(\left(A_{B}S\times N_{1}\right),\left(A_{D_{1}}\times N_{1}\right))}}+\underset{D_{2}}{\underline{\left(A_{D_{1}}\times N_{1}\right)}}\right\}. \end{array}$$

It should be mentioned that the duplexing loss is taken into account due to two-hop communication model. That is why, overall SE is divided by 1/2 in D2D-CRS-SM and 1/4 in VBLAST-ZF. This means that four time-slots in total are required to receive data by all the three users (NU, FU, and D${}_{2}$) if the considered network scenarios exploit VBLAST-ZF system, considering time-division multiple access. However, only two time slots are required in the proposed D2D-CRS-SM. Please note that for VBLAST-ZF, S${}_{1}$ and S${}_{2}$ are same.

## 5. Conclusions and Future Works

### 5.1. Conclusions

A D2D aided CRS exploiting SM (D2D-CRS-SM) considering two different scenarios for a downlink transmission was proposed and investigated, where each scenario consists of a BS, a NU, a FU, and a D2D transmitter (D${}_{1}$)-receiver (D${}_{2}$) pair. In the proposed D2D-CRS-SM, D${}_{1}$ cooperatively relays the traffic of a FU with no direct link with the BS. At the same time, D${}_{1}$ gets the opportunity to communicate with D${}_{2}$ without causing any mutual interference due to the exploitation of SM in D${}_{1}$. Moreover, no leasing of time slots or power allocation are required between the cellular and D2D user. The performance of D2D-CRS-SM is studied, in terms of BER, SE and sum SE, considering *M*-PSK and *M*-QAM. Moreover, a comparative study with VBLAST-ZF MIMO is also provided. The outcome of this study suggests that the proposed model can be a promising paradigm for the next generation wireless communications, where it is expected that cellular communication and D2D communication will coexist. It is worth mentioning that this is the basic model for a D2D aided CRS using SM.

### 5.2. Future Recommendations

Several possible improvements of this work can be possible, which can be the subject of future works. Firstly, a more general scenario with multiple NUs and FUs with the aid of multiple D2D pairs is anticipated to be studied. Secondly, hybrid DF- and AF-based cooperative D2D aided protocols can be investigated. Lastly, the proposed network models can be extended to the multi-cell scenario.

Although multiple technologies can be combined without too much effort in the simulation environment, there are many challenges to implement in a real environment. Some of them are as follows:

- There should be both technical and policy requirements agreement among different service providers. For example, in the proposed technique, the cellular users and D2D users should be in consensus for their mutual benefits.
- Whereas a simulation environment can assume perfect synchronization among the antennas or users and perfect channel estimation, the real environment introduces various challenging tasks.
- During software simulation, it is considered that software algorithm can be realized perfectly by hardware design. However, algorithm design is often bounded by the hardware condition in real environment [56].
- With the current 3rd generation partnership project (3GPP) cellular standards, different approaches need to be complied.

## Figures and Tables

**Table 1:** An example of mapping table at BS for data transmission during $P_{1}$. Transmission of 5-bits employing 2 antennas (A${}_{BS}$ = 2) and 16-QAM/16-PSK ($M_{1}$ = 16) or, 4 antennas (A${}_{BS}$ = 4) and 8-QAM/8-QPSK ($M_{1}$ = 8).

	A${}_{BS}$ = 2 and $M_{1}$ = 16		A${}_{BS}$ = 4 and $M_{1}$ = 8	
Input Bits,	Antenna	Transmit	Antenna	Transmit
$X_{1}$	Index/Number	Symbol	Index/Number	Symbol
00000	0	0000	00 (0)	000
00001	0	0001	00 (0)	001
00010	0	0010	00 (0)	010
00011	0	0011	00 (0)	011
00100	0	0100	00 (0)	100
00101	0	0101	00 (0)	101
00110	0	0110	00 (0)	110
00111	0	0111	00 (0)	111
01000	0	1000	01 (1)	000
01001	0	1001	01 (1)	001
01010	0	1010	01 (1)	010
01011	0	1011	01 (1)	011
01100	0	1100	01 (1)	100
01101	0	1101	01 (1)	101
01110	0	1110	01 (1)	110
01111	0	1111	01 (1)	111
10000	1	0000	10 (2)	000
10001	1	0001	10 (2)	001
10010	1	0010	10 (2)	010
10011	1	0011	10 (2)	011
10100	1	0100	10 (2)	100
10101	1	0101	10 (2)	101
10110	1	0110	10 (2)	110
10111	1	0111	10 (2)	111
11000	1	1000	11 (3)	000
11001	1	1001	11 (3)	001
11010	1	1010	11 (3)	010
11011	1	1011	11 (3)	011
11100	1	1100	11 (3)	100
11101	1	1101	11 (3)	101
11110	1	1110	11 (3)	110
11111	1	1111	11 (3)	111

**Table 2:** An example of mapping table at D${}_{1}$ for data transmission during $P_{2}$. For $N_{1}\left(=1\mathrm{and}2\right)$ bits of modulation symbol/antenna index from BS, D${}_{1}$ uses $2^{N_{1}}$-QAM/PSK.

	A${}_{D_{1}}$ = 8 and $N_{1}$ = 1		A${}_{D_{1}}$ = 4 and $N_{1}$ = 2	
Input Bits,	Antenna	Transmit	Antenna	Transmit
$X_{2}$	Index/Number	Symbol	Index/Number	Symbol
0000	000 (0)	0	00 (0)	00
0001	000 (0)	1	00 (0)	01
0010	001 (1)	0	00 (0)	10
0011	001 (1)	1	00 (0)	11
0100	010 (2)	0	01 (1)	00
0101	010 (2)	1	01 (1)	01
0110	011 (3)	0	01 (1)	10
0111	011 (3)	1	01 (1)	11
1000	100 (4)	0	10 (2)	00
1001	100 (4)	1	10 (2)	01
1010	101 (5)	0	10 (2)	10
1011	101 (5)	1	10 (2)	11
1100	110 (6)	0	11 (3)	00
1101	110 (6)	1	11 (3)	01
1110	111 (7)	0	11 (3)	10
1111	111 (7)	1	11 (3)	11

**Table 3:** Simulation Parameters.

SL#	NU [S${}_{1}$]	FU [S${}_{1}$]	D${}_{1}$→D${}_{2}$ [S${}_{1}$& S${}_{2}$]	NU [S${}_{2}$]	FU [S${}_{2}$]
1	A${}_{B}S$ = 2, A${}_{NU}$ = 2	$M_{1}$ = $M_{2}$ = 4, A${}_{B}S$ = 2, A${}_{D_{1}}$ = 2, A${}_{F}U$ = 2	A${}_{D_{1}}$ = 2, A${}_{D_{2}}$ = 2	$M_{1}$ = 4, A${}_{B}S$ = 2, A${}_{NU}$ = 2	$M_{2}$ = 2, A${}_{B}S$ = 2, A${}_{D_{1}}$ = 2, A${}_{FU}$ = 2
2	A${}_{B}S$ = 2, A${}_{NU}$ = 3	$M_{1}$ = $M_{2}$ = 16, A${}_{B}S$ = 2, A${}_{D_{1}}$ = 2, A${}_{F}U$ = 2	A${}_{D_{1}}$ = 2, A${}_{D_{2}}$ = 3	$M_{1}$ = 16, A${}_{B}S$ = 2, A${}_{NU}$ = 2	$M_{2}$ = 2, A${}_{B}S$ = 2, A${}_{D_{1}}$ = 2, A${}_{FU}$ = 3
3	A${}_{B}S$ = 2, A${}_{NU}$ = 4	$M_{1}$ = $M_{2}$ = 32, A${}_{B}S$ = 2, A${}_{D_{1}}$ = 2, A${}_{F}U$ = 2	A${}_{D_{1}}$ = 2, A${}_{D_{2}}$ = 4	$M_{1}$ = 32, A${}_{B}S$ = 2, A${}_{NU}$ = 2	$M_{2}$ = 2, A${}_{B}S$ = 2, A${}_{D_{1}}$ = 2, A${}_{FU}$ = 4
4	A${}_{B}S$ = 4, A${}_{NU}$ = 2	$M_{1}$ = $M_{2}$ = 4, A${}_{B}S$ = 4, A${}_{D_{1}}$ = 4, A${}_{F}U$ = 4	A${}_{D_{1}}$ = 4, A${}_{D_{2}}$ = 2	$M_{1}$ = 4, A${}_{B}S$ = 4, A${}_{NU}$ = 4	$M_{2}$ = 4, A${}_{B}S$ = 4, A${}_{D_{1}}$ = 4, A${}_{FU}$ = 2
5	A${}_{B}S$ = 4, A${}_{NU}$ = 3	$M_{1}$ = $M_{2}$ = 16, A${}_{B}S$ = 4, A${}_{D_{1}}$ = 4, A${}_{F}U$ = 4	A${}_{D_{1}}$ = 4, A${}_{D_{2}}$ = 3	$M_{1}$ = 16, A${}_{B}S$ = 4, A${}_{NU}$ = 4	$M_{2}$ = 4, A${}_{B}S$ = 4, A${}_{D_{1}}$ = 4, A${}_{FU}$ = 3
6	A${}_{B}S$ = 4, A${}_{NU}$ = 4	$M_{1}$ = $M_{2}$ = 32, A${}_{B}S$ = 4, A${}_{D_{1}}$ = 4, A${}_{F}U$ = 4	A${}_{D_{1}}$ = 4, A${}_{D_{2}}$ = 4	$M_{1}$ = 32, A${}_{B}S$ = 4, A${}_{NU}$ = 4	$M_{2}$ = 4, A${}_{B}S$ = 4, A${}_{D_{1}}$ = 4, A${}_{FU}$ = 4

**Table 4:** Maximum achievable theoretical sum SE comparison between D2D-CRS-SM and VBLAST-ZF.

Scenario	Antenna Configuration	D2D-CRS-SM	VBLAST-ZF
S${}_{1}$	A${}_{B}S$ = 4, A${}_{NU}$ = 4, A${}_{D_{1}}$ = 4, A${}_{F}U$ = 4, A${}_{D_{2}}$ = 4	Modulation order 4/16/32/64 -QAM: 3/4/4.5/5 bits/s/Hz	Modulation order 4-QAM: 6 bits/s/Hz
S${}_{2}$	A${}_{B}S$ = 4, A${}_{NU}$ = 4, A${}_{D_{1}}$ = 4, A${}_{F}U$ = 4, A${}_{D_{2}}$ = 4	Modulation order 4/16/32/64 -QAM at BS: 3/4/4.5/5 bits/s/Hz	Modulation order 4-QAM: 6 bits/s/Hz

## Appendix A. Analytical Symbol-Error-Rate Formulation

### Appendix A.1. Analytical Symbol-Error-Rate of FU in S_1_

In accordance with the concept of SM, both BS and D${}_{1}$ transmit FU data bits through the activation of a single antenna at a particular time instant. Therefore, we can consider the estimation of any transmitted *M*-PSK/*M*-QAM symbol at D${}_{1}$ and FU as a special case of $1\times A_{D_{1}}$ and $1\times A_{FU}$ MRC detection [12], respectively. Now by using [57] (Equation (41)), the error estimation of any transmitted *M*-PSK data symbol (SER) at D${}_{1}$ and FU in S${}_{1}$ can be obtained respectively as

(A1) $$\begin{array}{cc} P_{SER,FU}^{S_{1},P_{1}} & =\frac{1}{\pi }\int_{0}^{\frac{(M_{1}-1)\pi }{M_{1}}}\prod_{i=1}^{A_{D_{1}}}{\left(1+\frac{\rho g_{FU,1}}{\sin^{2}\phi }\right)}^{-1}d\phi \end{array}$$

(A2) $$\begin{array}{cc} P_{SER,FU}^{S_{1},P_{2}} & =\frac{1}{\pi }\int_{0}^{\frac{(M_{2}-1)\pi }{M_{2}}}\prod_{i=1}^{A_{FU}}{\left(1+\frac{\rho g_{FU,2}}{\sin^{2}\phi }\right)}^{-1}d\phi \end{array}$$

where $P_{SER,FU}^{S_{1},P_{1}}$ and $P_{SER,FU}^{S_{1},P_{2}}$ denote SER at D${}_{1}$ and FU during $P_{1}$ and $P_{2}$, respectively. Here, $g_{FU,1}=\sin^{2}\left(\frac{\phi }{M_{1}}\right)$, $g_{FU,2}=\sin^{2}\left(\frac{\phi }{M_{2}}\right)$, and $M_{2}=2^{N_{1}}$. The average SNR, ρ at the receiving side is denoted by $\rho ≜\frac{P_{BS}}{\sigma^{2}}≜\frac{P_{D_{1}}}{\sigma^{2}}$, where $\sigma^{2}$ is the noise variance.

Again by using [57] (Equation (41)), the error estimation of any transmitted *M*-QAM data symbol at D${}_{1}$ and FU in S${}_{1}$ can be obtained respectively as

(A3) $$\begin{array}{cc} P_{SER,FU}^{S_{1},P_{1}} & =\frac{4}{\pi }\left(1-\frac{1}{{\sqrt{M}}_{1}}\right)\int_{0}^{\frac{\pi }{2}}\prod_{i=1}^{A_{D_{1}}}{\left(1+\frac{\rho g_{QAM,1}}{\sin^{2}\phi }\right)}^{-1}d\phi \\ \; & -\frac{4}{\pi }{\left(1-\frac{1}{{\sqrt{M}}_{1}}\right)}^{2}\int_{0}^{\frac{\pi }{4}}\prod_{i=1}^{A_{D_{1}}}{\left(1+\frac{\rho g_{QAM,1}}{\sin^{2}\phi }\right)}^{-1}d\phi \end{array}$$

(A4) $$\begin{array}{cc} P_{SER,FU}^{S_{1},P_{2}} & =\frac{4}{\pi }\left(1-\frac{1}{{\sqrt{M}}_{2}}\right)\int_{0}^{\frac{\pi }{2}}\prod_{i=1}^{A_{FU}}{\left(1+\frac{\rho g_{QAM,2}}{\sin^{2}\phi }\right)}^{-1}d\phi \\ \; & -\frac{4}{\pi }{\left(1-\frac{1}{{\sqrt{M}}_{2}}\right)}^{2}\int_{0}^{\frac{\pi }{4}}\prod_{i=1}^{A_{FU}}{\left(1+\frac{\rho g_{QAM,1}}{\sin^{2}\phi }\right)}^{-1}d\phi \end{array}$$

where $g_{QAM,1}=\frac{3}{2(M_{1}-1)}$ and $g_{QAM,2}=\frac{3}{2(M_{2}-1)}$. Here, $P_{SER,FU}^{S_{1},P_{1}}$ and $P_{SER,FU}^{S_{1},P_{2}}$ denote SER at D${}_{1}$ and FU during $P_{1}$ and $P_{2}$, respectively.

### Appendix A.2. Analytical Symbol-Error-Rate of NU in S_2_

In a similar manner, we can also consider the estimation of any transmitted *M*-PSK/*M*-QAM symbol at NU in S${}_{2}$ as a special case of $1\times A_{NU}$ MRC detection [12] due to the exploitation of SM. Now the error estimation of any transmitted *M*-PSK data symbol at NU during $P_{1}$ in S${}_{2}$ can be obtained by using [57] (Equation (41)).

(A5) $$\begin{array}{cc} P_{SER,NU}^{S_{2},P_{1}} & =\frac{1}{\pi }\int_{0}^{\frac{(M_{1}-1)\pi }{M_{1}}}\prod_{i=1}^{A_{NU}}{\left(1+\frac{\rho g_{NU,1}}{\sin^{2}\phi }\right)}^{-1}d\phi \end{array}$$

where $P_{SER,NU}^{S_{2},P_{1}}$ denotes SER at NU during $P_{1}$ and $g_{NU,1}=\sin^{2}\left(\frac{\phi }{M_{1}}\right)$. The average SNR, ρ at the receiving side is denoted by $\rho ≜\frac{P_{BS}}{\sigma^{2}}$, where $\sigma^{2}$ is the noise variance.

Again by using [57] (Equation (41)), the error estimation of any transmitted *M*-QAM data symbol at NU during $P_{1}$ in S${}_{2}$ can be obtained as

(A6) $$\begin{array}{cc} P_{SER,NU}^{S_{2},P_{1}} & =\frac{4}{\pi }\left(1-\frac{1}{{\sqrt{M}}_{1}}\right)\int_{0}^{\frac{\pi }{2}}\prod_{i=1}^{A_{NU}}{\left(1+\frac{\rho g_{QAM,1}}{\sin^{2}\phi }\right)}^{-1}d\phi \\ \; & -\frac{4}{\pi }{\left(1-\frac{1}{{\sqrt{M}}_{1}}\right)}^{2}\int_{0}^{\frac{\pi }{4}}\prod_{i=1}^{A_{NU}}{\left(1+\frac{\rho g_{QAM,1}}{\sin^{2}\phi }\right)}^{-1}d\phi \end{array}$$

where $g_{QAM,1}=\frac{3}{2(M_{1}-1)}$ and $P_{SER,NU}^{S_{2},P_{1}}$ denotes SER at NU during $P_{1}$.
